# Sildenafil amplifies calcium influx and insulin secretion in pancreatic β cells

**DOI:** 10.14814/phy2.16091

**Published:** 2024-06-11

**Authors:** Naoya Murao, Risa Morikawa, Yusuke Seino, Kenju Shimomura, Yuko Maejima, Yuichiro Yamada, Atsushi Suzuki

**Affiliations:** ^1^ Department of Endocrinology, Diabetes and Metabolism School of Medicine, Fujita Health University Toyoake Japan; ^2^ Yutaka Seino Distinguished Center for Diabetes Research Kansai Electric Power Medical Research Institute Kyoto Japan; ^3^ Department of Bioregulation and Pharmacological Medicine School of Medicine, Fukushima Medical University Fukushima Japan

**Keywords:** diabetes, insulin secretion, pancreatic β cells, sildenafil, voltage‐dependent calcium channels

## Abstract

Sildenafil, a phosphodiesterase‐5 (PDE5) inhibitor, has been shown to improve insulin sensitivity in animal models and prediabetic patients. However, its other metabolic effects remain poorly investigated. This study examines the impact of sildenafil on insulin secretion in MIN6‐K8 mouse clonal β cells. Sildenafil amplified insulin secretion by enhancing Ca^2+^ influx. These effects required other depolarizing stimuli in MIN6‐K8 cells but not in K_ATP_ channel‐deficient β cells, which were already depolarized, indicating that sildenafil‐amplified insulin secretion is depolarization‐dependent and K_ATP_ channel‐independent. Interestingly, sildenafil‐amplified insulin secretion was inhibited by pharmacological inhibition of R‐type channels, but not of other types of voltage‐dependent Ca^2+^ channels (VDCCs). Furthermore, sildenafil‐amplified insulin secretion was barely affected when its effect on cyclic GMP was inhibited by PDE5 knockdown. Thus, sildenafil stimulates insulin secretion and Ca^2+^ influx through R‐type VDCCs independently of the PDE5/cGMP pathway, a mechanism that differs from the known pharmacology of sildenafil and conventional insulin secretory pathways. Our results reposition sildenafil as an insulinotropic agent that can be used as a potential antidiabetic medicine and a tool to elucidate the novel mechanism of insulin secretion.

## 1 INTRODUCTION

Glucose stimulates insulin secretion from pancreatic β cells in a multistep process. First, glucose metabolism increases intracellular ATP levels, which leads to ATP‐sensitive K^+^ (K_ATP_) channel closure, resulting in membrane depolarization and the opening of voltage‐dependent Ca^2+^ channels (VDCCs). This leads to an increase in intracellular Ca^2+^ (Ca^2+^_i_) and stimulation of insulin exocytosis (Henquin, 2009). Thus, glucose‐induced insulin secretion (GIIS) is primarily governed by the opening and closing of K_ATP_ channels in response to glucose metabolism. K_ATP_ channels comprise the pore‐forming subunit Kir6.2 encoded by *Kcnj11* and the sulfonylurea receptor SUR1 (Inagaki et al., 1995).

In type 2 diabetes, β cells cannot secrete sufficient insulin to counteract insulin resistance or excess nutrients, resulting in elevated blood glucose levels. Therefore, enhancement of insulin secretion from β cells is central to the treatment of diabetes. There are several options for clinical insulinotropic agents, including sulfonylureas, glinides, dipeptidyl peptidase‐4 inhibitors, and glucagon‐like peptide 1 analogs (American Diabetes Association Professional Practice Committee, 2024; Bouchi et al., 2022). Of these, sulfonylureas and glinides bind to SUR1, which in turn closes K_ATP_ channels and stimulates insulin secretion. This action is independent of blood glucose levels and, therefore, can cause hypoglycemia as a side effect. There is therefore a need for alternative therapeutic options.

Drug repurposing presents a promising solution for the development of novel insulinotropic medications. Sildenafil is a phosphodiesterase‐5 (PDE5) inhibitor primarily used for the treatment of erectile dysfunction and pulmonary arterial hypertension (Ghofrani et al., 2006). This drug has gained attention as a potential antidiabetic agent owing to its effectiveness in preclinical and clinical studies. Sildenafil has been reported to enhance insulin sensitivity in high‐fat diet‐fed mice (Ayala et al., 2007) and prediabetic patients (Ramirez et al., 2015). Additionally, sildenafil promotes vascular relaxation in diabetic rats (Schäfer et al., 2008) and is considered beneficial for treating vascular dysfunction in diabetic patients (Zimmermann et al., 2020).

However, the effect of sildenafil on insulin secretion from pancreatic β cells remains unclear. This study investigates the effect of sildenafil on insulin secretion using MIN6‐K8, a mouse clonal β cell line, as a model. Our results indicate that sildenafil promotes insulin secretion by increasing Ca^2+^ influx through R‐type VDCCs, and that this effect is not mediated by PDE5. This suggests a novel mechanism of action for sildenafil in β cells that differs from its known pharmacology.

## 2 MATERIALS AND METHODS

### 2.1 Cell lines

MIN6‐K8 cells were established as previously described (Iwasaki et al., 2010) and were kindly provided by Professor Junichi Miyazaki (Osaka University). *Kcnj11* ^*−/−*^ β cells (clone *Kcnj11* ^−/‐^βCL1) are β cells deficient in Kir6.2, established by sub‐cloning MIN6‐K8 cells transfected with Cas9 nickase and guide RNA pairs targeting mouse *Kcnj11*, as described previously (Oduori et al., 2020). All cells were cultured in Dulbecco's modified Eagle's medium (DMEM) containing 4500 mg/L glucose (Sigma‐Aldrich, St. Louis, MO, USA, Cat# D5796) supplemented with 10% fetal bovine serum (BioWest, Nuaillé, France, Cat# S1400‐500) and 5 ppm 2‐mercaptoethanol. The cells were maintained at 37°C with 5% CO_2_ and used for experiments in passages 22–30.

### 2.2 Reagents

Krebs‐Ringer bicarbonate buffer‐HEPES (133.4 mM NaCl, 4.7 mM KCl, 1.2 mM KH_2_PO_4_, 1.2 mM MgSO_4_, 2.5 mM CaCl_2_, 5 mM NaHCO_3_, 10 mM HEPES) containing 0.1% fatty acid‐free bovine‐serum albumin (Sigma‐Aldrich, St. Louis, MO, USA, Cat# A6003) and 2.8 mM glucose (2.8G‐KRBH) adjusted to pH 7.4 was used in insulin secretion and Ca^2+^ imaging experiments. Additional glucose (final concentration, 11.1 mM), sildenafil (Tokyo Chemical Industry, Tokyo, Japan, Cat# S0986), and glimepiride (Tokyo Chemical Industry, Tokyo, Japan, Cat# G0395) were added to KRBH during the stimulation period. Nifedipine (FUJIFILM Wako Pure Chemical, Osaka, Japan, Cat# 14505781), diazoxide (Tokyo Chemical Industry, Tokyo, Japan, Cat# D5402), SNX‐482 (Peptide Institute, Osaka, Japan, Cat# 4363‐s), ω‐conotoxin MVIIC (Peptide Institute, Osaka, Japan, Cat# 4283‐v), and thapsigargin (FUJIFILM Wako Pure Chemical, Osaka, Japan, Cat# 209‐17281) were added during the preincubation and stimulation periods. The reagents used for stimulation were stored as a 1000× concentrate in dimethyl sulfoxide (DMSO) (FUJIFILM Wako Pure Chemical, Osaka, Japan, Cat# 041‐29351) and diluted with KRBH shortly before the experiment. An equal volume of DMSO was added to the vehicle control. Ca^2+^‐free KRB was formulated by replacing CaCl_2_ with an equivalent concentration of MgCl_2_ and adding 0.2 mM EGTA (NACALAI TESQUE, Kyoto, Japan, Cat# 15214‐21).

### 2.3 Insulin secretion

Insulin secretion was measured using the static incubation method as described previously (Murao et al., 2022) with slight modifications. Briefly, cells of the same passage number were seeded in 24‐well plates (Corning, Glendale, AZ, USA, Cat# 353047) at a density of 5 × 10^5^ cells/well and cultured for 48 h. On the day of measurement, the cells were subjected to three successive washes with 2.8G‐KRBH, followed by a preincubation period of 30 min with 300 μL/well of 2.8G‐KRBH. Subsequently, the supernatant was replaced with 300 μL/well of fresh KRBH containing the specified stimulations and incubated for 30 min at 37°C.

The reaction was terminated by cooling the plate on ice for 10 min, after which the entire supernatant was collected for the quantification of released insulin using the homogeneous time‐resolved fluorescence assay Insulin Ultrasensitive kit (Revvity, Waltham, MA, USA, Cat# 62IN2PEH) in accordance with the manufacturer's instructions. Fluorescence was measured using an Infinite F Nano+ microplate reader (Tecan, Zürich, Switzerland).

### 2.4 Imaging of intracellular Ca^2+^

Cells were seeded in a 35‐mm glass‐bottom dish (Matsunami Glass, Osaka, Japan, Cat# D11530H) at a density of 1.28 × 10^5^ cells/dish and cultured for 48 h. Subsequently, the cells were loaded with 1 μM Fluo‐4 am (Dojindo, Kumamoto, Japan, Cat# F312) in 2.8G‐KRBH for 20 min at 37°C in room air. Following a brief washing, cells were loaded with 1 mL of fresh 2.8G‐KRBH and basal recordings were performed for 300 s (from time −300 to 0). Immediately after the addition of 1 mL KRBH supplemented with stimulations at 2× concentration, recordings were resumed for another 600 s (from time 0 to 600) with a time interval of 2 s.

Time‐lapse images were obtained using a Zeiss LSM 980 Airyscan2 inverted confocal laser scanning super‐resolution microscope equipped with a Plan Apo 40×, 1.4 Oil DICII objective lens (Carl Zeiss Microscopy, Jena, Germany). The cells were excited at 488 nm laser with 0.3% output power, and fluorescence emission was measured at 508–579 nm. During observation, the cells were maintained at 37°C using an incubator XLmulti S2 DARK (Pecon, Erbach, Germany).

Images were acquired in the frame mode at a rate of two frames per second and with an image size of 212.2 × 212.2 μm (512 × 512 pixels). The obtained images were analyzed using the ZEN 3.0 imaging software (Carl Zeiss Microscopy, Jena, Germany, RRID: SCR_021725). Cells were randomly chosen for analysis for each stimulation, and the number of cells analyzed is indicated in the figure legends. The fluorescence intensity of the entire cell body (*F*) was monitored and normalized to the average fluorescence intensity between −300 and 0 s (*F*0). The amplitude of Ca^2+^ responses was quantified as the incremental area under the curve (iAUC) using *F*/*F*0 = 1 as the baseline.

### 2.5 Knockdown of *Pde5a* using small interfering RNA (siRNA)

siRNAs targeting *Pde5a* (Dharmacon, Lafayette, CO, USA, Cat# M‐041115‐00‐0005) and non‐targeting siRNA (Dharmacon, Lafayette, CO, USA, Cat# D‐001206‐14‐50) were reverse‐transfected using the DharmaFECT 2 transfection reagent (Dharmacon, Lafayette, CO, USA, Cat# T‐2002‐03). Briefly, a complex of siRNA and DharmaFECT 2 was prepared in serum‐free DMEM (Sigma‐Aldrich, St. Louis, MO, USA, Cat# D5796) at a volume of 100 μL/well according to the manufacturer's instructions. Cells were resuspended in complete culture media at 1.25 × 10^6^ cells/mL. The cell suspension was then combined with the siRNA/DharmaFECT 2 complex. The final concentrations of siRNA and DharmaFECT 2 were 40 nM and 0.4%, respectively. For insulin secretion and RT‐qPCR experiments, the cells were seeded in 24‐well plates (Corning, Glendale, AZ, USA, Cat# 353047) at 5 × 10^5^ cells/500 μL/well. For immunoblotting, the cells were seeded in 12‐well plates (Corning, Glendale, AZ, USA, Cat# 353053) at 1 × 10^6^ cells/mL/well. For cGMP measurement, cells were seeded in 6‐well plates (Corning, Glendale, AZ, USA; Cat #353046) at 2 × 10^6^ cells/2 mL/well. The subsequent experiments were performed after a 48‐h culture.

### 2.6 RT‐qPCR

cDNA was prepared from 48‐h cultured cells using CellAmp Direct Lysis and RT set (Takara Bio, Shiga, Japan, Cat# 3737S/A) according to the manufacturer's instructions. Quantitative real‐time PCR was performed on a QuantStudio 7 Flex system (Thermo Fisher Scientific, Waltham, MA, USA, RRID:SCR_020245) using TaqMan Universal Master Mix II with UNG (Thermo Fisher Scientific, Waltham, MA, USA, Cat# 4440038) and TaqMan probes: *Pde5a* (Cat# Mm00463177_m1) and *Tbp* (Cat# Mm01277042_m1). Relative gene expression of *Pde5a* was calculated using the 2^−ΔΔCT^ method and normalized to *Tbp*.

### 2.7 Immunoblotting

MIN6‐K8 cells were transfected with siRNAs and cultured as described in Section 2.5. The cells were then lysed with 50 μL/well of lysis buffer (20 mM Tris–HCl pH 7.5, 150 mM NaCl, 1 mM EDTA, 1 mM EGTA, 1% NP‐40, 1% sodium deoxycholate, 0.1% SDS), and 1x complete protease inhibitor cocktail (Sigma‐Aldrich, St. Louis, MO, USA, Cat# 11697498001). The lysate was sonicated for 20 s on ice and centrifuged at 15,000 × *g* at 4°C for 10 min. The supernatant was collected and separated on a 7.5% polyacrylamide‐SDS gel and transferred to a PVDF membrane. The membranes were blocked with 3% bovine serum albumin (BSA) (Sigma‐Aldrich, St. Louis, MO, USA, Cat# A7906) in Tris‐buffered saline with Tween 20 (TBS‐T) and incubated with anti‐PDE5A antibody (1:100) (Santa Cruz Biotechnology, Dallas, TX, USA, Cat# sc‐398747) in TBS‐T supplemented with 3% BSA overnight at 4°C. The membrane was then incubated with HRP‐conjugated anti‐mouse immunoglobulins (1:2000) (Agilent, Santa Clara, CA, USA, Cat# P0447, RRID:AB_2617137) in TBS‐T supplemented with 1% BSA for 1 h at room temperature. Signals were visualized using ECL Prime detection reagent (Cytiva, Buckinghamshire, UK, Cat# RPN2232). Images were taken using ImageQuant 800 (Cytiva, Buckinghamshire, UK). Subsequently, the membrane was stripped using Restore Western Blot Stripping Buffer (Thermo Fisher Scientific, Waltham, MA, USA, Cat# 21059) for 15 min and re‐probed with anti‐α‐tubulin (1:1000) (Thermo Fisher Scientific, Waltham, MA, USA, Cat# A11126, RRID:AB_2534135). The images were quantified using ImageJ (version 1.53k, https://imagej.nih.gov/ij/index.html, RRID:SCR_003070).

### 2.8 cGMP measurement

MIN6‐K8 cells were transfected with siRNAs and cultured as described in Section 2.5. Cells were then rinsed three times with 2.8G‐KRBH, followed by preincubation with 2.8G‐KRBH for 60 min at 37°C. The supernatant was replaced with KRBH‐containing stimulations specified in Figure 4d, followed by incubation for another 30 min at 37°C, and the supernatant was discarded. Cells were quickly rinsed with ice‐cold water, extracted by the addition of 500 μL ice‐cold extraction buffer (67.5% methanol, 7.5% chloroform, and 25% water), and the whole plate was snap‐frozen in liquid nitrogen. Cells were thawed on ice, scraped into 2 mL screw tubes along with the supernatant, and stored at −80°C until extraction of the metabolites.

For extraction of the metabolites, samples were supplemented with 80 μL of diluted (1:640) internal standard (Human Metabolome Technologies, Yamagata, Japan, Cat# H3304‐1002), 165 μL of methanol, and 465 μL of chloroform. The samples were then homogenized using a pre‐cooled bead crusher at 3200 rpm for 1 min and centrifuged at 15,000 × *g* at 4°C for 3 min. The aqueous layer was transferred to pre‐wetted ultrafiltration tubes (Human Metabolome Technologies, Yamagata, Japan, Cat# UFC3LCCNB‐HMT) and centrifuged at 9100 × *g*, 4°C until completely filtrated. The filtrate was freeze‐dried, re‐dissolved in 10 μL of water, and subjected to mass spectrometry. The organic layer was evaporated by decompression at room temperature, and the residue was resuspended in lysis buffer (see Section 2.7), which was then subjected to a BCA protein assay (Thermo Fisher Scientific, Waltham, MA, USA, Cat# 23225).

cGMP was measured by G7100A capillary electrophoresis (Agilent Technologies, Santa Clara, CA, USA) interfaced with a G6224A time‐of‐flight LC/MS mass spectrometer (Agilent Technologies, Santa Clara, CA, USA). A G1310A isocratic pump (Agilent Technologies, Santa Clara, CA, USA) equipped with a G1379B degasser (Agilent Technologies, Santa Clara, CA, USA) was used to supply sheath liquid (Human Metabolome Technologies, Yamagata, Japan, Cat# H3301‐2020). The mass spectrometer was operated in the negative ionization mode. All separations were performed on fused silica capillaries (Human Metabolome Technologies, Yamagata, Japan, Cat# H3305‐2002) at 25°C using an anion analysis buffer (Human Metabolome Technologies, Yamagata, Japan, Cat# H3302‐2021) as the background electrolyte. The applied voltage was set to 30 kV at 20°C, together with a pressure of 15 mbar. Sheath liquid was delivered to a nebulizer by an isocratic pump at 1 mL/min. Chromatograms and mass spectra were analyzed by MassHunter qualitative analysis version 10.0 (Agilent Technologies, Santa Clara, CA, USA, RRID:SCR_015040). Annotation and quantification of chromatogram peaks were carried out using a standard mixture (Human Metabolome Technologies, Yamagata, Japan, Cat# 3032) as a reference.

### 2.9 Statistical analysis

Sample sizes were estimated from the expected effect size based on previous experiments. No randomization or blinding was used. For insulin secretion, RT‐qPCR, immunoblotting, and cGMP quantification, *n* represents the number of biological replicates of cells grown in different wells of the same multiwell plate. For Ca^2+^ measurements, *n* represents the number of different single cells analyzed. Data were shown as the mean ± standard deviation (SD) along with the plot of individual data points. For statistical comparisons between two groups, a two‐tailed unpaired Welch's unpaired *t*‐test was used. For comparisons between three groups, Welch's one‐way analysis of variance (ANOVA) was followed by pairwise comparisons corrected using Dunnett's method. Normality of the distribution was confirmed by the Shapiro–Wilk test. *p*‐values less than 0.05 were considered statistically significant and are indicated in the figures. *p*‐values more than 0.05 are not indicated in the figures. The statistical analyses used are indicated in the figure legends. Statistical analyses were performed using GraphPad Prism 9 (GraphPad Software, Boston, MA, USA, https://www.graphpad.com; RRID:SCR_002798).

## 3 RESULTS

### 3.1 Sildenafil amplifies insulin secretion from β cell lines

**FIGURE 1:**
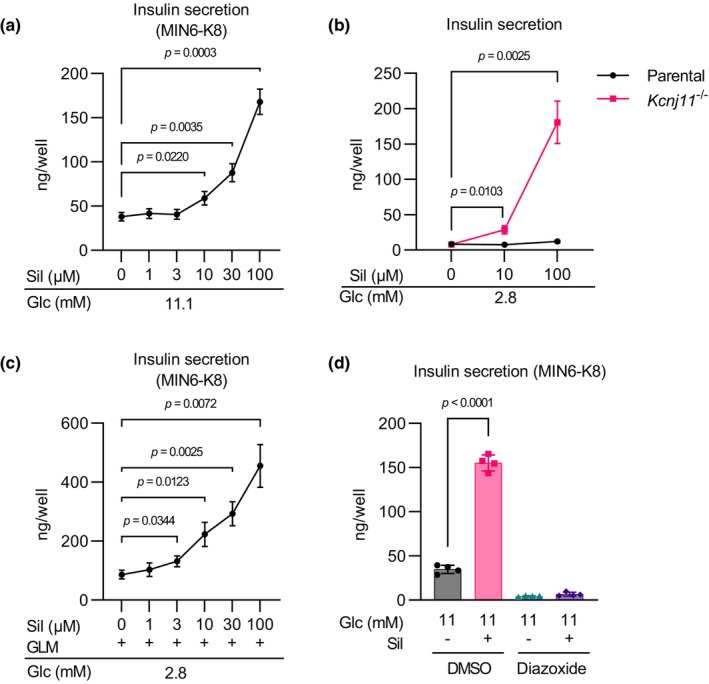
Sildenafil amplifies insulin secretion from β cell lines in a depolarization‐dependent manner. (a) Dose‐dependent effects of sildenafil on insulin secretion at 11.1 mM glucose in MIN6‐K8 cells. *n* = 4. (b) Effects of sildenafil on insulin secretion at 2.8 mM glucose in MIN6‐K8 and *Kcnj11* ^−/−^ β cells. *n* = 4. (c) Effect of sildenafil on insulin secretion in the presence of glimepiride at 2.8 mM glucose in MIN6‐K8 cells. *n* = 4. (d) Effect of diazoxide on sildenafil‐amplified insulin secretion in MIN6‐K8 cells. *n* = 4. Data were presented as mean ± standard deviation (SD). 2.8G, 2.8 mM glucose; 11.1G, 11.1 mM glucose. The reagents were added to achieve the following final concentrations unless otherwise specified: sildenafil (Sil)—100 μM, glimepiride (GLM)—1 μM, and diazoxide—100 μM. Statistical comparisons were performed using Welch's one‐way ANOVA with Dunnett's post hoc test in (a–c), and Welch's unpaired two‐tailed *t*‐test for (d).

The effect of sildenafil on insulin secretion was investigated using MIN6‐K8 cells. Sildenafil at concentrations of 10 μM or higher dose‐dependently enhanced insulin secretion at stimulatory levels (11.1 mM) of glucose (Figure 1a). Sildenafil at 10 or 100 μM had no effect at basal levels (2.8 mM) of glucose (Figure 1b), indicating that the insulinotropic effect of sildenafil is glucose‐dependent.

To assess whether sildenafil enhances insulin secretion by facilitating K_ATP_ channel closure, its efficacy was tested in K_ATP_ channel‐deficient β cells. We previously generated *Kcnj11* ^−/−^ β cells, in which K_ATP_ channel activity is absent and the cell membrane is depolarized continuously regardless of extracellular glucose levels (Oduori et al., 2020). Sildenafil at 10 and 100 μM dose‐dependently increased insulin secretion in *Kcnj11* ^−/−^ β cells even at 2.8 mM glucose (Figure 1b), demonstrating that sildenafil‐amplified insulin secretion is independent of K_ATP_ channel activity.

Similarly, in MIN6‐K8 cells, sildenafil at 3 μM or higher concentrations dose‐dependently amplified insulin secretion at 2.8 mM glucose in the presence of the sulfonylurea glimepiride, which inhibits K_ATP_ channel activity (Figure 1c). In contrast, sildenafil's insulinotropic effect at 11 mM glucose was lost in the presence of diazoxide (Figure 1d), a K_ATP_ channel opener that hyperpolarizes β cells (Gribble & Reimann, 2003; Rorsman & Ashcroft, 2018). These observations suggest that sildenafil is effective only when the plasma membrane is depolarized by other factors, such as high glucose, sulfonylureas, and *Kcnj11* knockout.

### 3.2 Sildenafil potentiates influx of extracellular Ca^2+^

We then investigated the association between sildenafil‐amplified insulin secretion and Ca^2+^_i_ using Fluo‐4 imaging.

**FIGURE 2:**
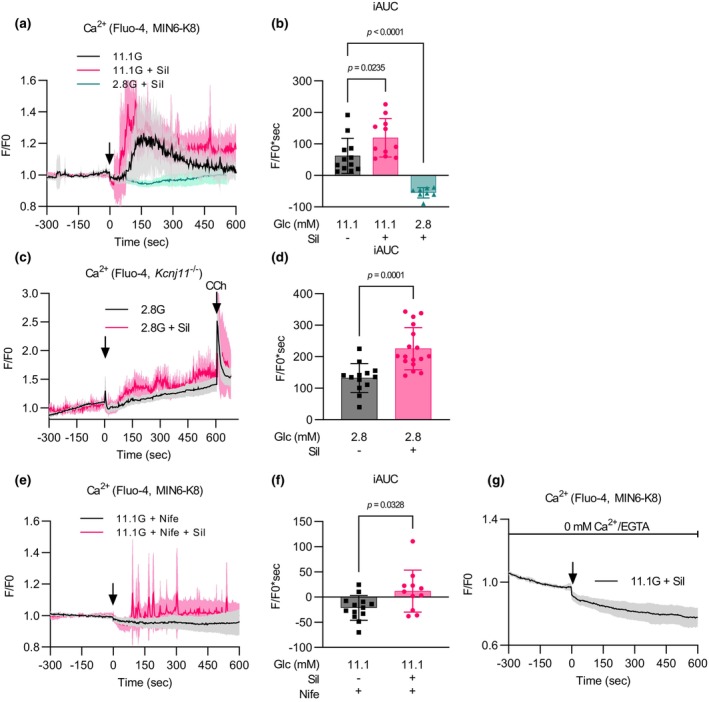
Sildenafil potentiates the influx of extracellular Ca^2+^. Intracellular Ca^2+^ levels were measured using Fluo‐4. The time course of normalized fluorescence intensity at 508–579 nm is indicated in (a), (c), (e), and (g). The black arrow indicates the addition of the indicated stimulations at time = 0. The magnitude of Ca^2+^ responses was quantified as iAUC in (b), (d), and (f). (a, b). Effect of sildenafil on intracellular Ca^2+^ in MIN6‐K8 cells. 2.8G + Sil: *n* = 8; 11.1G: *n* = 12; 11.1G + Sil: *n* = 11. (c, d) Effect of sildenafil on intracellular Ca^2+^ in *Kcnj11* ^−/−^ β cells. Carbachol was added at time = 600 as positive control. 11.1G + Nife: *n* = 13; 11.1G + Nife + Sil: *n* = 11. (e, f) Effect of nifedipine on sildenafil‐induced Ca^2+^ response in MIN6‐K8 cells. 11.1G + Nife: *n* = 13; 11.1G + Nife + Sil: *n* = 11. (g) Effect of sildenafil on intracellular Ca^2+^ under extracellular Ca^2+^‐free conditions in MIN6‐K8 cells. 2.8G + Sil: *n* = 8; 11.1G: *n* = 12; 11.1G + Sil: *n* = 11. Data are presented as mean ± SD. SD is indicated by shaded regions in (a), (c), (e), and (g), and by error bars elsewhere. 2.8G, 2.8 mM glucose; 11.1G, 11.1 mM glucose. The reagents were added to achieve the following final concentrations: sildenafil (Sil)—100 μM, nifedipine (Nife)—10 μM, carbachol (CCh)—50 μM, and EGTA—0.2 mM. Statistical comparisons were made using Welch's one‐way ANOVA with Dunnett's post hoc test in (b), and Welch's unpaired two‐tailed *t*‐test for (d) and (f).

In MIN6‐K8 cells, sildenafil augmented the increase in Ca^2+^_i_ induced by 11.1 mM glucose but had no apparent effect at 2.8 mM glucose (Figure 2a,b). In *Kcnj11* ^−/−^ β cells, sildenafil increased Ca^2+^_i_ levels at 2.8 mM glucose (Figure 2c,d). These trends accord with the pattern of insulin secretion shown in Figure 1, suggesting that sildenafil‐amplified insulin secretion involves augmented Ca^2+^_i_ responses.

**FIGURE 3:**
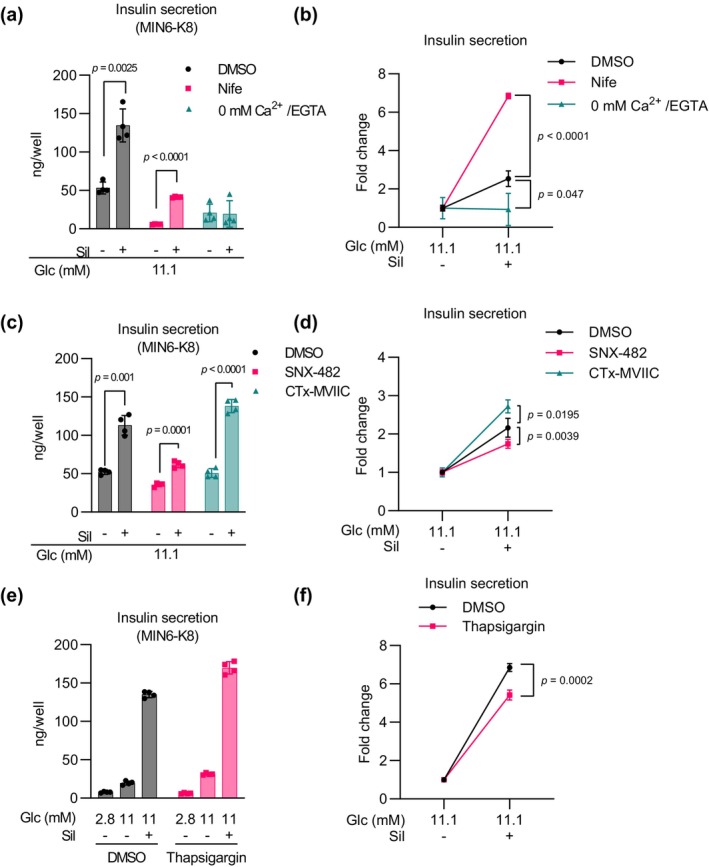
Sildenafil‐amplified insulin secretion is dependent on Ca^2+^ influx through R‐type VDCCs. (a, b) Effects of nifedipine or extracellular Ca^2+^‐free conditions on sildenafil‐amplified insulin secretion. *n* = 4. The data are presented in their original value in (a) and as fold change over 11.1 mM glucose in (b). (c, d) Effects of SNX‐482 and conotoxin (CTx)‐MVIIC on sildenafil‐amplified insulin secretion. *n* = 4. The data are presented in their original value in (c) and as fold change over 11.1 mM glucose in (d). (e, f) Effect of thapsigargin on sildenafil‐amplified insulin secretion. *n* = 4. The data are presented in their original value in (e) and as fold change over 11.1 mM glucose in (f). All experiments were performed using MIN6‐K8 cells. Data were presented as mean ± SD. The reagents were added to achieve the following final concentrations: sildenafil (Sil)—100 μM, nifedipine (Nife)—10 μM, EGTA—0.2 mM, SNX‐482—500 nM, CTx‐MVIIC—2.5 μM, and thapsigargin—1 μM. Statistical comparisons were performed using Welch's unpaired two‐tailed *t*‐test for (a), (c), and (f), and Welch's one‐way ANOVA with Dunnett's post hoc test for (b) and (d).

Nifedipine, an inhibitor of L‐type VDCCs, eliminated the response to 11.1 mM glucose but did not completely inhibit the response to a combination of sildenafil and 11.1 mM glucose (Figure 2e,f). This combination maintained a higher baseline and produced multiple spikes in Ca^2+^ traces even in the presence of nifedipine (Figure 2e). These responses were abolished upon removal of Ca^2+^ from the stimulation buffer (Figure 2g). Consistently, nifedipine treatment substantially lowered insulin secretion by 11.1 mM glucose compared to vehicle alone but did not block the responsiveness to sildenafil (Figure 3a), which was increased as expressed by fold change (Figure 3b). In contrast, Ca^2+^‐free buffer abrogated sildenafil responsiveness (Figure 3a,b). These results indicate that sildenafil‐induced Ca^2+^_i_ response and insulin secretion involve nifedipine‐insensitive extracellular Ca^2+^ influx. We tested the involvement of non‐L‐type VDCCs using their inhibitors. SNX‐482 inhibits R‐type VDCC with high selectivity (Newcomb et al., 1998). ω‐Conotoxin MVIIC inhibits N‐ and P/Q‐type VDCCs (Gurkoff et al., 2013). Although neither of these inhibitors completely suppressed sildenafil responsiveness (Figure 3c), SNX‐482 decreased and ω‐Conotoxin MVIIC increased sildenafil responsiveness as expressed by fold change (Figure 3d). These findings suggest that R‐type VDCCs mediate sildenafil‐amplified Ca^2+^_i_ response.

We also assessed the role of intracellular Ca^2+^ stores using thapsigargin, an inhibitor of sarcoplasmic/endoplasmic reticulum Ca^2+^‐ATPase (SERCA), to deplete intracellular Ca^2+^ stores. Thapsigargin only marginally affected insulin secretion, with a slight decrease in sildenafil responsiveness as measured by the fold change (Figure 3e,f). This result indicates that intracellular Ca^2+^ stores are dispensable for the sildenafil‐induced Ca^2+^_i_ response.

### 3.3 Sildenafil‐amplified insulin secretion is independent of PDE5

**FIGURE 4:**
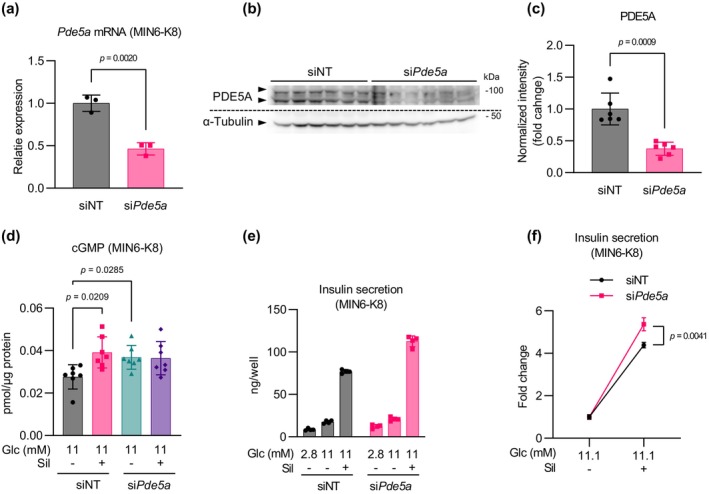
PDE5 is not involved in sildenafil‐amplified insulin secretion. (a) Knockdown efficiency of *Pde5a* assessed by RT‐qPCR. mRNA levels were normalized to siNT (non‐targeting siRNA)‐treated cells. *n* = 3. (b, c) Knockdown efficiency of PDE5A protein assessed by immunoblotting. Two PDE5A isoforms (isoforms 1 and 2) were detected and are indicated by black triangles. The average intensity of the two PDE5A isoforms was normalized to α‐tubulin and expressed as fold change in (c). *n* = 6. (d). Effect of *Pde5a* knockdown on the regulation of intracellular cGMP by sildenafil. Whole‐cell cGMP levels were normalized to total protein. *n* = 7. (e, f) Effect of *Pde5a* knockdown on sildenafil‐amplified insulin secretion. *n* = 4. The data are presented in their original value in (e) and as fold change over 11.1 mM glucose in (f). All experiments were performed using MIN6‐K8 cells. Data were presented as mean ± SD. Sildenafil (Sil)—100 μM. Statistical comparisons were made using Welch's unpaired two‐tailed *t*‐test for (c) and (f), and Welch's one‐way ANOVA with Dunnett's post hoc test for (d).

We next investigated whether PDE5, the original molecular target of sildenafil, plays a role in its insulinotropic effect. Using siRNA to knock down *Pde5a*, we successfully decreased its transcript and protein levels by approximately 50% (Figure 4a–c). *Pde5a* knockdown elevated basal cGMP levels, impeding the ability of sildenafil to further increase cGMP levels (Figure 4d). However, under this condition, there was no suppression of sildenafil‐amplified insulin secretion, which actually appeared to increase, as expressed by the fold change (Figure 4e,f). These findings indicate that the insulinotropic effect of sildenafil is independent of PDE5 inhibition.

## 4 DISCUSSION

In the present study, we show that (1) sildenafil‐amplified insulin is dependent on depolarization but independent of K_ATP_ channels; (2) Sildenafil enhances insulin secretion by augmenting extracellular Ca^2+^ influx through R‐type VDCCs; and that (3) sildenafil‐amplified insulin secretion is not mediated by the PDE5‐inhibitory effects of sildenafil.

This is the first report of a direct effect of sildenafil on pancreatic β‐cells and suggests its favorable therapeutic properties as an insulinotropic agent in the treatment of diabetes. Research has suggested that K_ATP_ channel activity is impaired in diabetic β cells (Nichols et al., 2022; Oduori et al., 2020). Unlike sulfonylureas and glinides, which are ineffective in K_ATP_ channel‐inactivated β cells, sildenafil remains effective. In addition, the insulinotropic effect of sildenafil is dependent on glucose levels, thereby reducing the likelihood of hypoglycemia as a potential side effect. Moreover, the beneficial effects of sildenafil on insulin sensitivity (Ayala et al., 2007; Ramirez et al., 2015) and vascular function (Schäfer et al., 2008; Zimmermann et al., 2020) might well complement its insulinotropic effect to further improve glycemic control.

The nature of sildenafil‐induced Ca^2+^ influx is intriguing because it involves R‐type VDCCs. In mouse β cells, glucose‐induced Ca^2+^ influx is predominantly mediated by L‐type VDCCs (Rorsman & Ashcroft, 2018; Thompson & Satin, 2021). Thus, treatment with DHP such as isradipine and nifedipine, or genetic ablation of CaV1.2, a subunit of L‐type VDCC, profoundly suppresses GIIS (Schulla et al., 2003). However, there is also a DHP‐insensitive component in GIIS, which is attributable to R‐, P/Q‐, and possibly N‐type VDCCs in mouse β cells (Davalli et al., 1996; Rorsman & Ashcroft, 2018; Thompson & Satin, 2021). Although R‐type VDCCs are required for the second phase of GIIS in mice (Jing et al., 2005), these channels have not been detected in human β cells (Braun et al., 2008). Therefore, the effectiveness of sildenafil should be validated in human islets.

The known pharmacology of sildenafil involves increased intracellular cyclic guanosine monophosphate (cGMP) levels through inhibition of its hydrolysis via PDE5. Elevated cGMP levels activate protein kinase G (PKG), leading to various cellular responses. Indeed, research has suggested that cGMP/PKG activation can enhance insulin secretion through PKG‐mediated K_ATP_ channel inhibition (Ropero et al., 1999) or membrane depolarization by unidentified K^+^ channels (Ishikawa et al., 2003). In addition, sildenafil can increase Ca^2+^_i_ influx by cGMP/PKG‐dependent activation of Ca^2+^‐activated K^+^ channels with large conductance (BK channels) in human umbilical vein endothelial cells (HUVEC) (Luedders et al., 2006). However, these pathways do not seem to be involved in sildenafil‐amplified insulin secretion, insofar as *Pde5a* knockdown, which impeded the ability of sildenafil to boost cGMP levels, enhanced sildenafil responsiveness, indicating that sildenafil‐amplified insulin secretion and cGMP/PKG signaling are not correlated.

This study has several limitations. First, the results are based solely on immortalized clonal β cells, which may not accurately represent the function of primary β cells in vivo. Further confirmation using primary islets or β cells is warranted. Second, this study did not confirm its findings in vivo. While previous studies failed to observe any change in plasma insulin or C‐peptide levels after chronic sildenafil administration in diet‐induced obese mice (Ayala et al., 2007; Johann et al., 2018) or prediabetic humans (Ramirez et al., 2015), this lack of effect may be attributed to the pharmacological profile of sildenafil. Whereas sildenafil's serum concentration in vivo is typically less than 1 μM (474.6 ng/mL) after a single dose, as determined by pharmacokinetic analysis (Alwhaibi et al., 2021; Nichols et al., 2002), the drug must be present at concentrations greater than approximately 10 μM (4.746 μg/mL) to stimulate insulin secretion. Therefore, to bridge the gap between in vitro and in vivo studies, the administration protocol must be optimized to achieve effective concentrations. Importantly, elevated sildenafil concentrations may cause cardiovascular and other adverse effects. No clear relationship has been established between serum sildenafil concentration and clinical toxicology. For example, 22.2 μg/mL (46.7 μM) sildenafil caused only tachycardia and vomiting in one case (Matheeussen et al., 2015), while 6.27 μg/mL (13.2 μM) and lower concentrations have been reported in fatal cases (Al Ibrahim et al., 2022; Tracqui et al., 2002). Caution must be taken when repositioning sildenafil as an insulinotropic agent to prevent toxicity.

## 5 CONCLUSION

Our results indicate that sildenafil increases insulin secretion by enhancing Ca^2+^ influx via R‐type VDCCs independently of PDE5. This study presents a new perspective on the metabolic advantages of sildenafil and provides insights into the molecular mechanism of insulin secretion.

## AUTHOR CONTRIBUTIONS

*Conceptualization*: Naoya Murao. *Methodology*: Naoya Murao. *Investigation*: Naoya Murao, Risa Morikawa, Kenju Shimomura, and Yuko Maejima. *Writing—Original draft*: Naoya Murao and Risa Morikawa. *Writing—Review and Editing*: Naoya Murao, Risa Morikawa, Kenju Shimomura, Yuko Maejima, Yusuke Seino, Yuichiro Yamada, and Atsushi Suzuki. *Data curation*: Naoya Murao and Risa Morikawa. *Visualization*: Naoya Murao and Risa Morikawa. *Supervision*: Kenju Shimomura, Yusuke Seino, Yuichiro Yamada, and Atsushi Suzuki. *Funding acquisition*: Naoya Murao and Atsushi Suzuki.

## FUNDING INFORMATION

This study was supported by Japan Society for the Promotion of Science (JSPS) KAKENHI Grant Numbers JP22K20869 and JP23K15401 for N.M. Research grants for N.M. were provided by the Japan Association for Diabetes Education and Care, Daiwa Securities Foundation, Suzuken Memorial Foundation, Japan Diabetes Foundation—Nippon Boehringer Ingelheim Co., Ltd. The Hori Sciences and Arts Foundation, Manpei Suzuki Diabetes Foundation, and Fujita Health University.

## CONFLICT OF INTEREST STATEMENT

The authors declare no conflict of interest.

## ETHICS STATEMENT

Our work only used culture cell lines. Therefore, this article does not contain any data relevant to ethics approval statement.

## Data Availability

Data supporting the findings of this study are available from the corresponding author upon reasonable request.
